# Case Report: Recurrent Autoimmune Hypoglycemia Induced by Non-Hypoglycemic Medications

**DOI:** 10.3389/fimmu.2022.855350

**Published:** 2022-07-22

**Authors:** Qiuping Zhu, Hanxin Zhao, Wei Qiu, Fang Wu, Chungen Qian, Yonghong Yang, Ye Kang, Fenping Zheng, Jiaqiang Zhou

**Affiliations:** ^1^ Department of Endocrinology and Metabolism, Sir Run Run Shaw Hospital, Zhejiang University School of Medicine, Hangzhou, China; ^2^ Department of Endocrinology, Haining People’s Hospital, Haining, China; ^3^ Department of Endocrinology, Huzhou Central Hospital, Huzhou, China; ^4^ College of Life Science and Technology, Huazhong University of Science and Technology, Wuhan, China; ^5^ Reagent R&D Center, Shenzhen YHLO Biotech Co., Ltd, Shenzhen, China

**Keywords:** hypoglycemia, insulin autoimmune syndrome, insulin autoantibodies, clopidogrel, case report

## Abstract

We present a case of recurrent autoimmune hypoglycemia induced by non-hypoglycemic agents. We review reported cases of autoimmune hypoglycemia related to non-hypoglycemic agents, and discuss the effects of different detection methods for insulin autoantibodies on the results obtained. We aim to provide information for clinicians and a warning for medication usage. Considering the increasing number of clopidogrel-induced AIH cases and the hypoglycemia-induced increase in the risk of cardiovascular events, we recommend that cardiovascular disease patients being treated with clopidogrel be informed of this rare side effect and that clinicians be vigilant for the possibility of autoimmune hypoglycemia in this patient population.

## Introduction

Autoimmune hypoglycemia (AIH) or insulin autoimmune syndrome (IAS) is a rare condition characterized by recurrent hypoglycemia, hyperinsulinemia, and positive insulin autoantibodies (IAAs). AIH was first reported by Hirata et al. in 1970 and is also called Hirata’s disease (1). AIH-associated hypoglycemia has a spontaneous and irregular onset, and varies in severity, duration, and remission rates (2). The underlying etiology is IAA formation triggered by autoimmune diseases, sulfhydryl drugs, or insulin use (2). We report a case of recurrent AIH caused by non-hypoglycemic agents.

## Case Description

A 76-year-old woman presented with a 3-year history of recurrent palpitations, hand tremors, and sweating with worsening of these symptoms since 1 month. The symptoms usually occurred with hunger. During severe episodes, she had abnormal behavior and confusion. Her venous blood glucose levels during the episodes were 1.4–2.8 mmol/L. The symptoms were relieved by eating or intravenous glucose. The patient had been examined at a regional hospital 2 years ago. A 75-g oral glucose tolerance test and insulin–C-peptide release test showed an extremely high serum insulin level along with a low blood glucose level, which indicated endogenous hyperinsulinemia (**Table 1**). However, a qualitative IAA test (immunoblot assay; Blot Biotech, Shenzhen, China) was negative. Tests for antinuclear antibody profile, immunoglobulins (IgG, IgM, and IgA), and complements (C3 and C4) were negative. The hemoglobin A1c level was 5.7%. The levels of growth hormone, insulin-like growth factor-1, thyroid hormones, reproductive hormones, and cortisol were within their reference ranges. Blood and urine ketones were negative. Enhanced abdominal magnetic resonance imaging and positron-emission tomography-computed tomography showed no significant findings. She had a history of hypertension and coronary heart disease without a history of thyroid disease, malignant tumor, or diabetes. She had never been exposed to hypoglycemic agents or exogenous insulin, neither did her cohabitants. Since the cause of the hypoglycemia was unclear, she was transferred to another hospital.

**Table 1:** Results of oral glucose tolerance tests and insulin–C-peptide release tests.

First hospitalization				
Time(hour)	Glucose(mmol/L)	Insulin^1^(2.6–23 μIU/mL)	C-peptide(1.1–4.4 ng/mL)	
0	2.94	> 1000	6.56	
1	6.36	> 1000	11.74	
2	8.82	> 1000	14.21	
3	11.4	> 1000	20.67	
4	5.62	> 1000	16.91	
5	1.27	> 1000	11.97	
**Second hospitalization**				
**Time** **(hour)**	**Glucose** **(mmol/L)**	**Insulin^1^** **(μIU/mL)**	**Insulin^2^** **(μIU/mL)**	**C-peptide** **(ng/mL)**
0	5.4	488.1	20.34	6.71
2	11.5	> 1000	100.5	20.6

^1^Insulin tested using the chemiluminescence method

^2^Insulin tested after 30% polyethylene glycol precipitation

At the second hospital, her insulin level was found to be significantly elevated (245 μIU/mL; chemiluminescence method) during hypoglycemia. However, the free insulin concentration detected after polyethylene glycol precipitation was much lower (**Table 1**). A qualitative IAA test was negative (same kit as above). A diagnosis of AIH with an unclear cause was considered. She was administered 4 mg methylprednisolone tablets three times a day for 1 week, but the hypoglycemia still recurred. She was then treated with 80 mg methylprednisolone injection daily for 6 days, which provided some relief and considerably decreased the insulin and C-peptide levels. The injections were replaced with 12 mg methylprednisolone tablets, which were gradually tapered and discontinued within approximately 1 month. Follow-up tests revealed a fasting insulin level of 56.72 μIU/mL and a C-peptide level of 3.38 ng/mL. The hypoglycemia stopped after this treatment.

**Figure 1:**
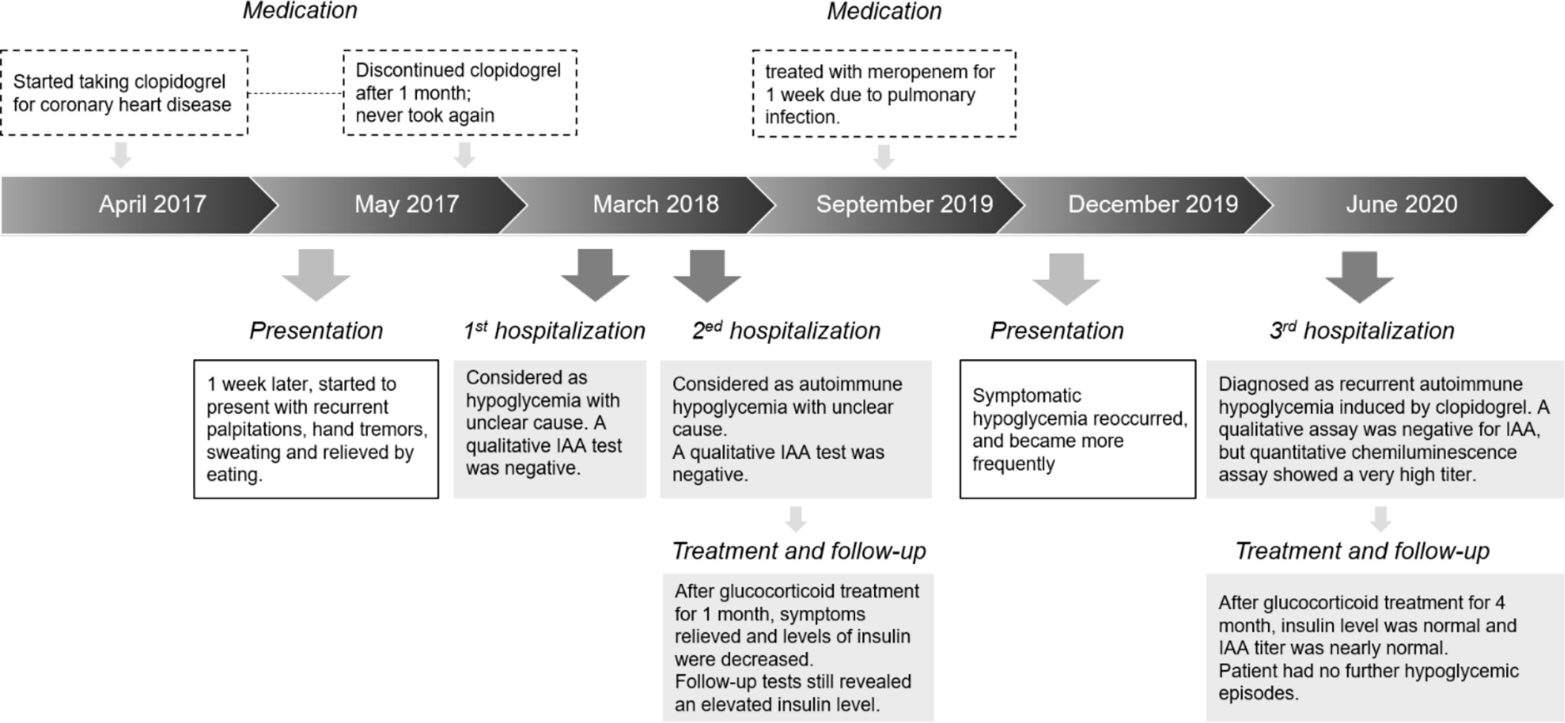
Timeline of Medical Event.

Six months before the current admission, the hypoglycemia recurred, and the insulin and C-peptide levels again increased. Months later, the patient frequently experienced symptomatic hypoglycemia, with sporadic palpitations, hand tremors, sweating, and unbearable hunger, which were obvious at night and before meals, and relieved by eating food. Her peripheral blood glucose levels during hypoglycemia were 2.1–3.4 mmol/L. A detailed medication history revealed that 3 years ago, she started taking clopidogrel for coronary heart disease and atrial fibrillation 1 week before the first hypoglycemic symptoms; these tablets were discontinued 1 month later. Nine months ago, three months before hypoglycemia recurrence, she was treated with meropenem for 1 week due to an infectious fever (Timeline shown in **Figure 1**). After admission to our hospital, laboratory examinations revealed the following: random blood glucose, 2.4 mmol/L; plasma insulin, >300 μIU/mL (1.9–23 μIU/mL); and C-peptide, 11.6 ng/mL (1.1–4.4 ng/mL). Interestingly, a qualitative immunoblot assay (same kit as above) was still negative for IAAs, but a quantitative chemiluminescence assay (Shenzhen YHLO Biotech, Shenzhen, China) showed a very high IAA titer of 61.8 cutoff index (COI), its results were interpreted as follows: COI < 0.9, non-reactive; COI ≥ 0.9 to < 1.1, indeterminate; and COI ≥ 1.1, reactive. We therefore conducted a further IAA subtype analysis, which identified IgG1 and IgG3 as the main subtypes. In addition, electrochemiluminescence was used to verify this finding, and the result was strongly positive. Localizing studies showed no evidence of insulinoma. Other related examinations showed no obvious abnormalities. Considering the medical history, we diagnosed AIH, and treated the patient with oral methylprednisolone starting at 4 mg twice a day; this treatment reduced the frequency of hypoglycemia and gradually reduced the insulin, C-peptide, and IAA levels even as the dosage was reduced. After 4 months of treatment, the insulin and C-peptide levels were normal, and the IAA titer was 3 COI. The patient had no further hypoglycemic episodes.

## Discussion

In this report, a non-diabetic patient presented with recurrent autonomic nervous excitation symptoms (palpitations, hand tremors, and sweating), hunger, and neurological glucose deficiency symptoms (abnormal behavior and confusion). During symptomatic phases, her blood glucose level was <2.8 mmol/L. The symptoms were relieved by eating or glucose infusion, which is consistent with the Whipple triad, confirming hypoglycemia. The plasma insulin and C-peptide levels were significantly increased during hypoglycemia. The insulin/C-peptide molar ratio was > 1, indicating endogenous hyperinsulinemia (3). The insulin recovery rate decreased significantly after polyethylene glycol precipitation, suggesting the presence of insulin-antibody complexes (3, 4). Although a qualitative IAA test was negative, AIH was still considered. Glucocorticoid treatment relieved the hypoglycemia and sharply decreased the insulin and C-peptide values, but did not result in normal insulin levels.

During the current hospitalization, the patient’s insulin level was significantly increased, and a quantitative test showed an elevated IAA titer; the diagnosis of AIH was clear. A detailed review of the patient’s history revealed that just 1 week before the first hypoglycemic symptom, she had taken clopidogrel tablets. Although clopidogrel does not contain sulfhydryl groups, its metabolites do (5). Therefore, it was considered to have induced AIH. The cause of the second recurrence was more difficult to determine. The patient had received meropenem for 1 week 3 months before the recurrence. Although no case of meropenem-induced AIH has been reported to date, its chemical structure is similar to that of imipenem (6), which is known to cause AIH. Moreover, both drugs contain sulfhydryl groups, which can induce hypoglycemia. In addition, after the first course of methylprednisolone, the insulin level decreased but remained high, suggesting that the clopidogrel-induced IAAs were not completely eliminated. Thus, the recurrence might be ascribed to residual IAAs or a meropenem-triggered amplified immune response.

The onset time of drug-induced AIH varies greatly, from days to months and even years after the drug exposure (2, 7–10). On average, the onset time is 4–6 weeks (11, 12). Many cases of AIH caused by non-hypoglycemic drugs are self-limiting. On average, spontaneous remission occurs within 3–6 months (13). Persistent recurrent hypoglycemia can last for 2.1–21.9 years (14).

The key to AIH treatment is cessation of the responsible agent. Another important part is dietary management. Small, frequent, low-carbohydrate meals are helpful (15). Medical treatments include acarbose, somatostatin analogues, diazoxide, glucocorticoids, azathioprine, and rituximab (2). Prednisone is the most common choice; the initial dose is 30–60 mg/d, and this is gradually reduced until the IAA test is negative, which can take 2 weeks to 1 year (16). Methylprednisone and dexamethasone can also be used (17). In the case of refractory hypoglycemia, plasmapheresis may rapidly reduce the IAA titer (18), and it has also been successfully treated with rituximab to deplete B lymphocytes (19).

IAA testing is necessary for AIH diagnosis. Qualitative immunoblot assay and quantitative chemiluminescence assay are commonly used detection methods in clinical practice. In some cases, the results of the two methods may be inconsistent. For our patient, the IAA titer during hypoglycemia was significantly increased according to the chemiluminescence assay but negative according to immunoblotting. This may be explained as follows: (1) The two kits use insulin antigens derived from different sources; the YHLO kit uses recombinant human insulin as the antigen, while the immunoblot assay uses native insulin from a bovine source, which has some differences to human insulin (20, 21). (2) Both assays could only detect the IgG type of IAAs, but the YHLO chemiluminescence kit could detect four IgG subtypes. Thus, patients with certain IgG subtypes may have false-negative results with the immunoblot assay. (3) The different methods use different antigen coatings and reaction systems. In the chemiluminescence method, a recombinant antigen is biotinylated with small molecular biotin and combined with streptavidin-coated paramagnetic particles. This method has a unique antigen, unique secondary antibody and substrate, and unique reaction environment and conditions. The antigen-antibody reaction is more efficient, thereby achieving the best reaction effect and leading to antibody detection. The immunoblot method uses a mixed protein antigen that is extracted from bovine pancreatic cells, which includes insulin, glutamic acid decarboxylase, and islet cell antigen. In addition, the antigen is directly coated onto a nitrocellulose membrane. So, it may be difficult to achieve a sufficient reaction for each detection item (20–22).

AIH is a hyperinsulinemic hypoglycemia associated with insulin antibodies. Its pathogenesis is believed to be associated with autoimmune deficiency or specific drug-induced IAAs against a susceptible genetic background. The relationship between some non-hypoglycemic drugs and AIH has been confirmed. Most of these drugs or their active metabolites contain sulfhydryl groups. These groups interact with the disulfide bond of insulin molecules, causing structural changes in insulin, which triggers an immune response and leads to IAA production. Insulin antibodies are characterized by low affinity and high volume. As blood glucose levels rise after meals, normally secreted insulin binds to IAAs, affecting insulin use (2). When the blood glucose concentration decreases, the insulin-antibody complex will dissociate spontaneously, releasing a large amount of active free insulin and causing hypoglycemia (23). We searched the PubMed and CNKI databases for related studies published between January 1970 and July 2021, by using the key words “insulin autoimmune syndrome, Hirata’s disease, and autoimmune hypoglycemia”. Cases of AIH associated with non-hypoglycemic agents are listed in **Table 2**. The top three drugs were methimazole, α-lipoic acid, and tiopronin (24). Clopidogrel was the fifth most common drug if adding our case (5, 17, 18, 24–26). Moreover, 11 of the 134 AIH cases with unknown causes were considered to have been caused by clopidogrel tablets, as each of these patients had a history of exposure to clopidogrel or a history of coronary heart disease or coronary or carotid artery stent implantation (19, 27–31). Therefore, the incidence of clopidogrel-caused AIH might be much higher than is currently believed. This may be explained by the low awareness of non-hypoglycemic agent-induced AIH.

**Table 2:** Classes of medications related to AIH and number of cases reported.

Classification	Medication	Cases
Antithyroid drugs	Methimazole Propylthiouracil Carbimazole	178 (126) 5 (3) 8 (1)
Health supplements	α-Lipoic acid Coenzyme Q10 Pyritinol	39 (11) 2 (1) 4 (2)
Antiplatelet drugs	Clopidogrel	9 (3)
Antihypertensive drugs	Captopril Metoprolol Diltiazem Procainamide Enalapril Hydralazine Metoprolol	23 (19) 1 (0) - - - 2 (0) 1 (0)
Hepatoprotective medicine	Reduced glutathione Tiopronin	4 (2) 37 (10)
For erectile dysfunction	Sildenafil	1 (0)
Antirheumatic drugs	Gold glucosamine	3 (0)
Antibiotics	Isoniazid d-Penicillamine Imipenem Penicillin G	2 (0) 6 (1) 1 (0) 1 (0)
Mucolytic agent	Acetylcysteine	2 (2)
Anti-inflammatory analgesics	Gu ci xiao zeng pill Loxoprofen sodium	1 (1) 1 (0)
Proton pump inhibitors	Pantoprazole Omeprazole Esomeprazole	2 (1) 1 (0) 1 (1)
Muscle relaxants	Thiocolchicoside Tolperisone	1 (0) 1 (0)
Antidepressants	Sertraline	1 (0)
Plasma proteins	Albumin	1 (0)
Antineoplastics	Tumor necrosis factor-inhibitor Aceglatone Interferon alfa	9 (0) 1 (0) 2 (0)
Chinese traditional medicine	Unknown ingredients	2 (2)
Amino acid	Methionine	2 (0)
Anti-inflammatory	Steroid	1 (0)
Unknown cause		134 (4)

-: The relevant review reported that the drug can cause autoimmune hypoglycemia, but no specific study was found in this search. The numbers in brackets are the cases reported in the Chinese literature.

Clopidogrel plays an important role in the treatment of cardio-cerebrovascular diseases caused by high platelet aggregation. It is the first-line choice for patients with atherosclerotic cardiovascular diseases. Cardiovascular disease patients with hypoglycemia face higher risks of cardiovascular events. Therefore, healthcare providers caring for patients using clopidogrel should be aware of the rare but serious side effect of autoimmune hypoglycemia. If such symptoms occur, timely blood glucose testing and sugar intake are necessary. We suggest that AIH be included as a rare but serious side effect of clopidogrel in clinical medication guides.

## Data Availability Statement

The raw data supporting the conclusions of this article will be made available by the authors, without undue reservation.

## Ethics Statement

The studies involving human participants were reviewed and approved by Sir Run Run Shaw Hospital, Zhejiang University School of Medicine. The patients/participants provided their written informed consent to participate in this study. Written informed consent was obtained from the individual(s) for the publication of any potentially identifiable images or data included in this article.

## Author Contributions

QZ - treating the patient and writing the article. HZ - treating the patient and writing the article. WQ - clinical data collection and analysis. FW - clinical data analysis and article review. CQ - testing and analysis of insulin autoantibody subtype. YY - testing and analysis of insulin autoantibody subtype. YK - testing and analysis of insulin autoantibody subtype. FZ - treating the patient and reviewing the article. JZ - treating the patient, writing and reviewing the article. All authors contributed to the article and approved the submitted version.

## Conflict of Interest

Author CQ, YY and YK were employed by Shenzhen YHLO Biotech Co., Ltd.

The remaining authors declare that the research was conducted in the absence of any commercial or financial relationships that could be construed as a potential conflict of interest.

## Publisher’s Note

All claims expressed in this article are solely those of the authors and do not necessarily represent those of their affiliated organizations, or those of the publisher, the editors and the reviewers. Any product that may be evaluated in this article, or claim that may be made by its manufacturer, is not guaranteed or endorsed by the publisher.

## Abbreviations

AIH, autoimmune hypoglycemia; IAS, insulin autoimmune syndrome; IAA, insulin autoantibody.
